# Presence and function of stress granules in atrial fibrillation

**DOI:** 10.1371/journal.pone.0213769

**Published:** 2019-04-03

**Authors:** Guo Dong, Fengying Liang, Bo Sun, Chengcheng Wang, Yangyang Liu, Xiangpeng Guan, Bo Yang, Chunhong Xiu, Ning Yang, Fengyu Liu, Tianyi Lu, Wei Han

**Affiliations:** 1 Department of Cardiology, The First Affiliated Hospital of Harbin Medical University, Harbin, People's Republic of China; 2 Department of Cardiology, The Third Affiliated Hospital of Guangzhou Medical University, Guangzhou, People's Republic of China; 3 Department of Cardiovascular surgery, The First Affiliated Hospital of Harbin Medical University, Harbin, People's Republic of China; Medical Faculty Mannheim, University of Heidelberg, GERMANY

## Abstract

**Aims:**

Stress granules (SGs) are transient cytoplasmic mRNA and protein complexes that form in eukaryotic cells under stress. SGs are related to multiple diseases, but there are no reports of the existence of SGs in atrial fibrillation (AF).

**Methods and results:**

Cell models of AF were established by field stimulation at 600 times per minute. HL-1 cells, cardiomyocytes and cardiac fibroblasts were transfected with G3BP1-cDNA plasmid by Lipofectamine 2000. The presence of SGs was detected by immunofluorescence analysis against GTPase-activating protein SH3 domain binding protein 1 (G3BP1) and poly(A)-binding protein 1 (PABP-1) and electron microscopy. Stable HL-1 cell lines transfected with lentivirus overexpressing G3BP1were constructed to further induce the formation of SGs in AF. Reactive oxygen species (ROS) and calcium overload in tachypaced HL-1 cells were studied by flow cytometry. The effects of G3BP1 overexpression on cardiac fibroblast proliferation and the protein expression level of collagen I/III and fibronectin-1 were also studied. Additionally, we detected protein synthesis in general and in single cells by puromycin incorporation in paced HL-1 cells. Here, we first showed that SGs are present in both tachypaced mouse cardiomyocytes and HL-1 atrial cells, although the presence is partial and at a low level. G3BP1 overexpression promoted SG formation, inhibited the rapid pacing-induced increase in ROS level, and attenuated calcium overload in HL-1 cells (all *P*<0.05). Furthermore, G3BP1 overexpression inhibited cardiac fibroblast proliferation (*P*<0.05) and decreased the protein expression level of collagen I and fibronectin-1 in cardiac fibroblasts stimulated by angiotensin II (all *P*<0.05). The bulk puromycin incorporation analyzed by Western blot did not show a global reduction in protein synthesis. However, puromycin incorporation in single cells detected by immunofluorescence analysis showed that protein synthesis in SG-containing cells significantly reduced (*P*<0.01).

**Conclusions:**

SGs are rapidly induced and present partially in AF, and G3BP1 overexpression promotes SG formation and confers cytoprotection against oxidative stress, calcium overload and atrial fibrosis in AF.

## Introduction

In response to a stress environment, such as oxidative stress, heat shock, ultraviolet radiation and viral infection, one of the main defense mechanisms of eukaryotic cells is the formation of stress granules (SGs). Cells either activate protective mechanisms or initiate apoptosis, depending on the type and level of stress [1]. SGs are cytoplasmic contents that lack an envelope and are not present under normal growth conditions but can be induced by stress stimulation. SGs typically include untransformed mRNAs, small 40S ribosomal subunits, mRNA-associated translation initiation complexes and many RNA-binding proteins, some of which are specific to SGs, such as Ras-GAP SH3-binding protein (G3BP1) [2]. A lack of these unique proteins will block the formation of SGs [2]. Previous studies have shown that the composition of the SGs is not immutable, and SGs have distinct components under different stress conditions; for example, SGs induced by heat shock contain HSP27, which is not present in the granules induced by arsenite [3]. A recent study also suggested that approximately 20% of SG diversity is stress- or cell-type-dependent [4].

The exact function of SGs has always been a topic of debate in many articles. In most cases, SGs are proposed to protect endogenous mRNA from stress; however, SGs represent more than just the dynamic sorting of mRNA between translation and decay. SGs also perform other cellular functions [5]. G3BP1 is considered a nucleating RNA-binding protein and play a crucial role in the aggregation of SGs [6,7]. By gene knockout or overexpression, G3BP1 and its binding partner, ubiquitin-specific protease 10 (USP10), have been shown to have antioxidant activity and to synergistically regulate the antioxidant activity of SGs [8]. Upon exposure to arsenic, an oxidative stress inducer, USP10 is recruited into SGs, thus reducing reactive oxygen species (ROS) production and inhibiting ROS-dependent apoptosis [8,9]. Moreover, USP10 interacts with many proteins localized at polysomes to control the stability and/or translation of mRNA(s) involved in redox control [10, 11]. In addition, recent studies have shown that SGs play an important role in neurodegenerative diseases [11,12]. However, there are still no reports of the existence of SGs in atrial fibrillation (AF).

AF is the most common arrhythmia in clinical practice and is an independent risk factor for stroke complications in cardiovascular and cerebrovascular diseases [13]. At present, the most extensive theory of the pathophysiology and pathogenesis of AF is that inflammation and oxidative stress affect atrial electrical and structural remodeling, thereby increasing the duration and recurrence of AF [14–16]. The rapid pacing of cells is equivalent to a strong oxidative stimulus, which can directly lead to pathophysiological changes, including electrical remodeling of AF [14,15], and may thus induce the formation of SGs. SGs are increasingly implicated in human neurodegeneration. However, SG behavior in cardiomyocytes during AF is much less known. Here, we show that SGs marked by G3BP1 and PABP1 present in tachypaced HL-1 and primary cardiomyocytes. G3BP1 overexpression can significantly reduce ROS and calcium overload in tachypaced HL-1 cardiomyocytes and prevent cardiac fibroblast proliferation and collagen formation in cardiac fibroblasts. In conclusion, we were the first to demonstrate the partial existence of SGs in cardiomyocytes and the protective role of G3BP1 in AF.

## Materials and methods

### HL-1 cell culture, transfection and pacing

HL-1 cells were obtained from mouse atrium, donated by Professor William Claycomb (Louisiana State University, New Orleans, LA, USA). The cells were cultured at 37°C in a humidified atmosphere of 5% CO2. Experiments were performed using cells between passages 150 and 200. The complete medium consisted of 87% Claycomb medium (Sigma-Aldrich 51800C), 10% fetal bovine serum (FBS, Sigma- Aldrich F2442), 1% glutamine (2 mM) and norepinephrine (100 μM norepinephrine in 30 mM L-ascorbic acid), and 1% penicillin/streptomycin (100 U/mL: 100 μg/mL). HL-1 cells were transfected with pcDNA and G3bP1-cDNA plasmid (Shanghai Genechem Co., LTD, China) for 72 h by Lipofectamine 2000 (Life Technologies, USA) in 6-well plates. Cell models of AF were established by field stimulation at 600 times per minute (10 Hz/5 ms, 1.0 V/cm; YC-2-S programmed stimulator, Chengdu Instrument Factory, China) as in our previous study [17].

### Cardiomyocyte and cardiac fibroblast culture and treatment

Cardiomyocytes and cardiac fibroblast were isolated from 1- to 3-day-old SD rats. All animal experimental procedures were approved by the ethics committee of the First Affiliated Hospital of Harbin Medical University and conformed to published NIH guidelines for animal research (NIH Publication No. N01-OD-4–2139). After anesthetized with pentobarbital sodium (50 mg/kg), rats were sacrificed by cervical dislocation, and then hearts were rapidly excised. Preplating was used for cardiomyocyte and cardiac fibroblast separation. In detail, the upper third of the hearts were finely minced in Dulbecco’s modified Eagle medium (DMEM, Gibco) containing 10% FBS. Pancreatin (Gibco, USA) was added into the heart tissue sample for digestion at 37°C. The supernatant was transferred into culture medium (DMEM containing 10% FBS) on ice. The above steps were repeated until tissues were completely digested. Then cells were filtered and centrifuged at 1000 rpm for 5 minutes. The supernatant was carefully poured off and then the final pellet was suspended in a medium containing 10% fetal bovine serum. The content was plated in a flask at 37°C and 5% of CO_2_ for fibroblasts adhesion. Cardiomyocytes were isolated from adherent fibroblasts after incubation for 1.5 h. BRDU with a final concentration of 0.1 mmol/L was added to further purify cardiomyocytes. Cardiomyocytes were used for experiments after 8 days of extraction and the fibroblasts were harvested while 2 times of the passages.

Cardiomyocytes and cardiac fibroblasts were transfected with pcDNA or G3bP1-cDNA plasmid (Shanghai Genechem Co., LTD, China) for 72 h by Lipofectamine 2000 (Life Technologies, USA). Cardiomyocytes were paced with field stimulation at 600 times per minute as mentioned above. Angiotensin II (AngII, 1 μM) was used to stimulate cardiac fibroblasts, and cardiac fibroblast proliferation was assessed with Cell Counting Kit-8 (Sigma Aldrich) according to the instructions. The cell proliferation was measured with absorbance at 450 nm.

### Construction of a stable cell line

To determine the function of SGs, cell lines stably expressing G3BP1 were constructed. First, the lowest lethal concentration of puromycin was determined by testing concentrations of 0 μg/mL, 0.5 μg/mL, 1.0 μg/mL, 1.5 μg/mL, 2.0 μg/mL, and 3.0 μg/mL. A selection medium containing 1 μg/mL puromycin was prepared. HL-1 cells were seeded in 24-well plates, and adherent cells were infected with virus particles containing pEGFP-LV-G3BP1 and pEGFP-LV-con. The ratio of the number of cells expressing GFP, as observed under a fluorescence microscope, to the total number of cells under a light microscope was calculated. The effect of puromycin on cell growth was determined, and whether to continue dosing was decided. When the percentage of cells with green fluorescence was above 98% after screening, a stable cell line was obtained.

### Immunofluorescence staining to detect SGs

Cells were fixed with 4% paraformaldehyde, washed with PBS, permeabilized with 0.1% Triton X-100, and then incubated in a blocking solution (Goat serum, Beyotime Biotechnology) for 0.5 h. Cells were then incubated with anti-G3BP1 (Abcam, USA) and/or anti-PABP1 (Abcam, USA) antibodies overnight at 4°C. After a PBS wash, the cells were incubated with an Alexa Fluor 546-conjugated antibody (Molecular Probes, USA) for 1 h. The stained cells on coverslips were washed three times with PBST. Nuclei were stained with DAPI (Sigma, USA) at room temperature. Cells were imaged with a Carl Zeiss Axio VertA1 microscope (Carl Zeiss Microimaging, Thornwood, USA).

### Levels of ROS and Ca^2+^ detected by flow cytometry

HL-1 cell samples were incubated with 10 μM DCFH-DA (Sigma-Aldrich, USA) at 37°C for ROS detection and were loaded with Fluo 3/AM (5 μM) (Beyotime Biotechnology, China) at 37°C to measure the content of Ca^2+^ within HL-1 cells. The fluorescence signal was measured at an excitation wavelength of 488 nm and emission wavelength of 525 nm with flow cytometry (BD FACSCantoII).

### Western blot analysis

The protein concentration in the supernatant was determined by BCA assay. Aliquots of 60 μg protein were separated by SDS-PAGE and transferred onto nitrocellulose membranes by an electric transfer process (BIORAD Inc., USA). The membranes were blocked and incubated with primary antibodies, including anti-G3BP1 (Abcam, USA), anti-COLI (Proteintech Group Inc., China), anti-COLIII (Proteintech Group Inc., China), anti-FN1 (Proteintech Group Inc., China), and anti-GAPDH (Beijing Zhongshan Biotechnology Co., China) antibodies. The blots were then incubated with horseradish peroxidase-conjugated antibodies (Beijing Zhongshan Jinqiao Biotechnology Co., China). Images were captured on an Odyssey Infrared Imaging System (LI-COR Biosciences, USA) and quantified using Odyssey v1.2.

### General protein synthesis

To detect general protein synthesis, puromycin incorporation was used in paced HL-1 cells. The cells were paced for 6h, then 10 μg/mL of puromycin (Solarbio, China) was added into the cell culture medium for 1 hour. The total protein was extracted, and the puromycin monoclonal (Merck millipore, USA) was used, and the incorporation level of puromycin was detected by Western blot. In addition, the concentration of protein and DNA was measured in HL-1 cells. Simply, HL-1 cells were lysed with cell lysate (Beyotime biotechnology, China) on ice for 15 min, and then centrifuged at 13500 rpm. The total protein concentration in the supernatant was measured using a BCA kit (Beyotime biotechnology, China). DNA was extracted in a centrifugal column by a DNA kit (Tiangen biotech CO., China), and then the concentration was determined. The ratio of protein to DNA was calculated.

### Protein synthesis in single cells

To assess the protein synthesis rate in single cells, puromycin incorporation was measured by immunofluorescence analysis. After paced for 6h, HL-1 cells were added with 10 μg/mL of puromycin (Solarbio, China) for 1 hour. Some cells were pretreated with 355 μM cycloheximide (BioVision, USA), a translation inhibitor, for 15 minutes serving as a negative control. Cells were fixed with 4% paraformaldehyde and allowed to stand at room temperature for 30 min. After washed with PBS, they were permeabilized with 0.1% Trion-X100 and blocked with 5% skim milk. The cells were incubated with primary antibody against puromycin (Merck millipore, USA) for 4 h, and then primary antibody against G3BP1 (Abcam, USA) overnight. Subsequently, the cells were incubated with goat anti-mouse secondary antibodies (Beijing Zhongshan Biotechnology Co., China) for 1 h, followed by staining with DAPI for 15 min. The images were captured by a laser confocal microscope (Zeiss LSM 510, Germany) with an excitation wavelength of 358 nm and emission 461 nm.

### Electron microscopy

Electron microscopy was performed in HL1 cells after they were paced for 6 hours. In detail, HL-1 cells adhered to a six-well plate were digested with 0.25% trypsin-EDTA solution, collected in a centrifuge tube and centrifuged at 2000 rpm for 5 minutes. The supernatant was discarded, and the cells were fixed with 2.5% glutaraldehyde at 4°C for 1–3 h. Dehydration was performed with an alcohol gradient; then, the cells were dried first with acetonitrile followed by vacuum drying. Samples were observed under the microscope after carbon spray and gold spray.

### Statistical analysis

The results are expressed as the mean ± SEM. Multiple-group comparisons were conducted by ANOVA. After ANOVA, if the results are significant, Bonferroni (LSD) was used to analyze the significance between two groups. All P values were 2 tailed. Values of P<0.05 were considered statistically significant. SPSS version 17.0 was used for all statistical evaluations.

## Results and discussion

### SGs exist in both tachypaced HL-1 cell lines and primary cardiomyocytes

**Fig 1:**
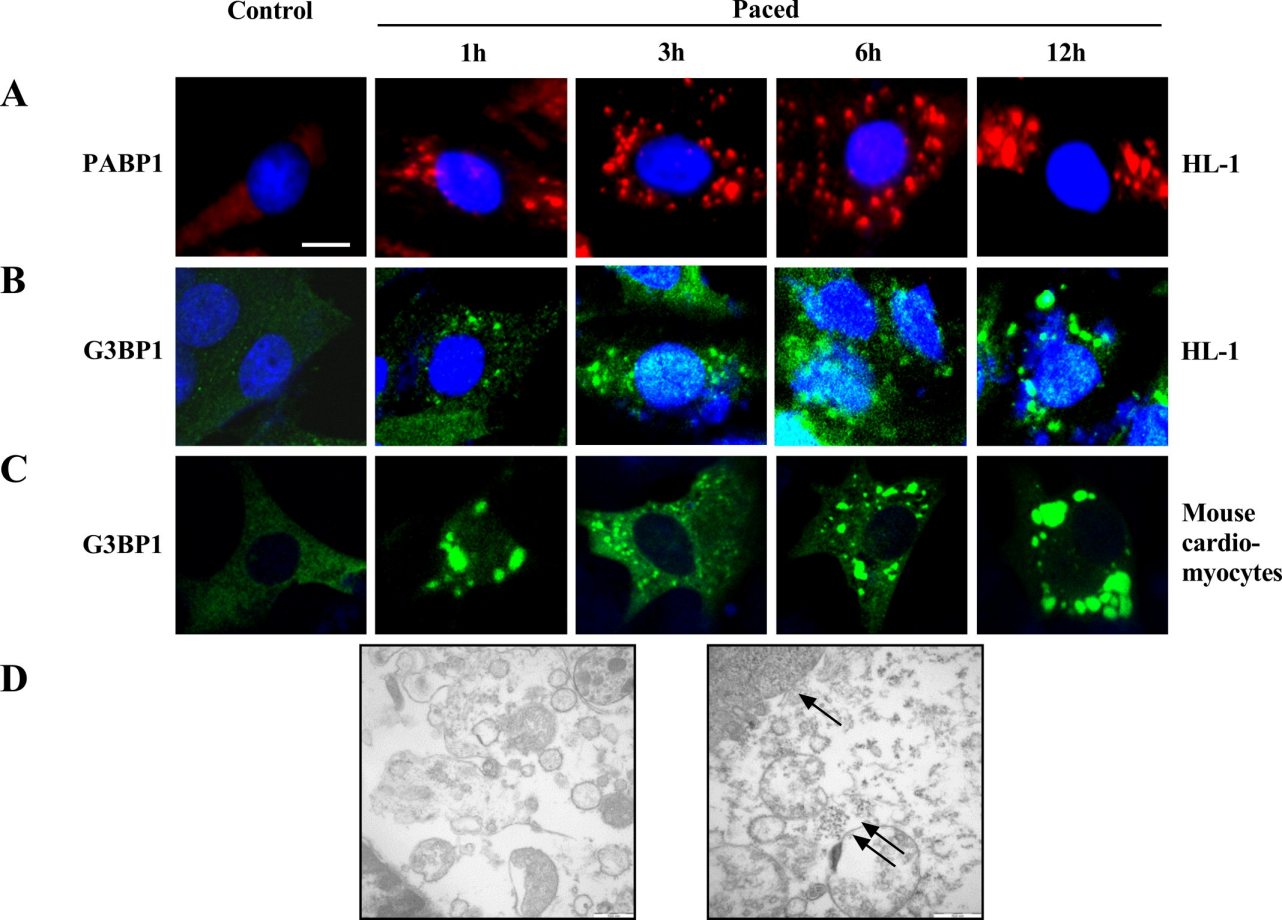
SGs exist in tachypaced HL-1 cells and atrial myocytes. (A,B) Endogenous SGs was probed with anti-PABP1 antibody (A) and anti-G3BP-1 antibody (B) in paced HL-1 cells at different time points. (C) Cardiomyocytes isolated from newborn SD rats were transfected with green fluorescent pEGFP-C1-G3BP1 plasmid and then paced. (D) The presence of SGs in atrial myocytes without and with tachypacing was confirmed by electron microscopy (scale bar 30 μm, ↑indicates the nucleus and ↑↑indicates the SGs).

We detected the expression of endogenous PABP-1 protein by red fluorescent antibody and exogenous G3BP1 by green fluorescent antibody to locate SGs. The presence of SGs was not observed in normal HL-1 cells and primary cardiomyocytes without stimulation (Fig 1A 0 h). However, SGs were observed at 1 h after pacing as large particles scattered in the cytoplasm. With increased stimulus time, the number of SGs increased gradually, and the particle size decreased; in addition, the SGs were eventually distributed uniformly in the cytoplasm (Fig 1A 3 h, 6 h). After pacing for 12 h, small SGs fused into large particles, and the number of particles decreased significantly (Fig 1A–1C). Thus, we confirmed that rapid pacing induced the formation of SGs in both HL-1 atrial myocytes (Fig 1A and 1B) and primary cardiomyocytes isolated from newborn SD rats (Fig 1C). The presence of SGs in tachypaced atrial myocytes was also confirmed by electron microscopy (Fig 1D).

### Exogenous G3BP1 overexpression was used to increase SG formation

**Fig 2:**
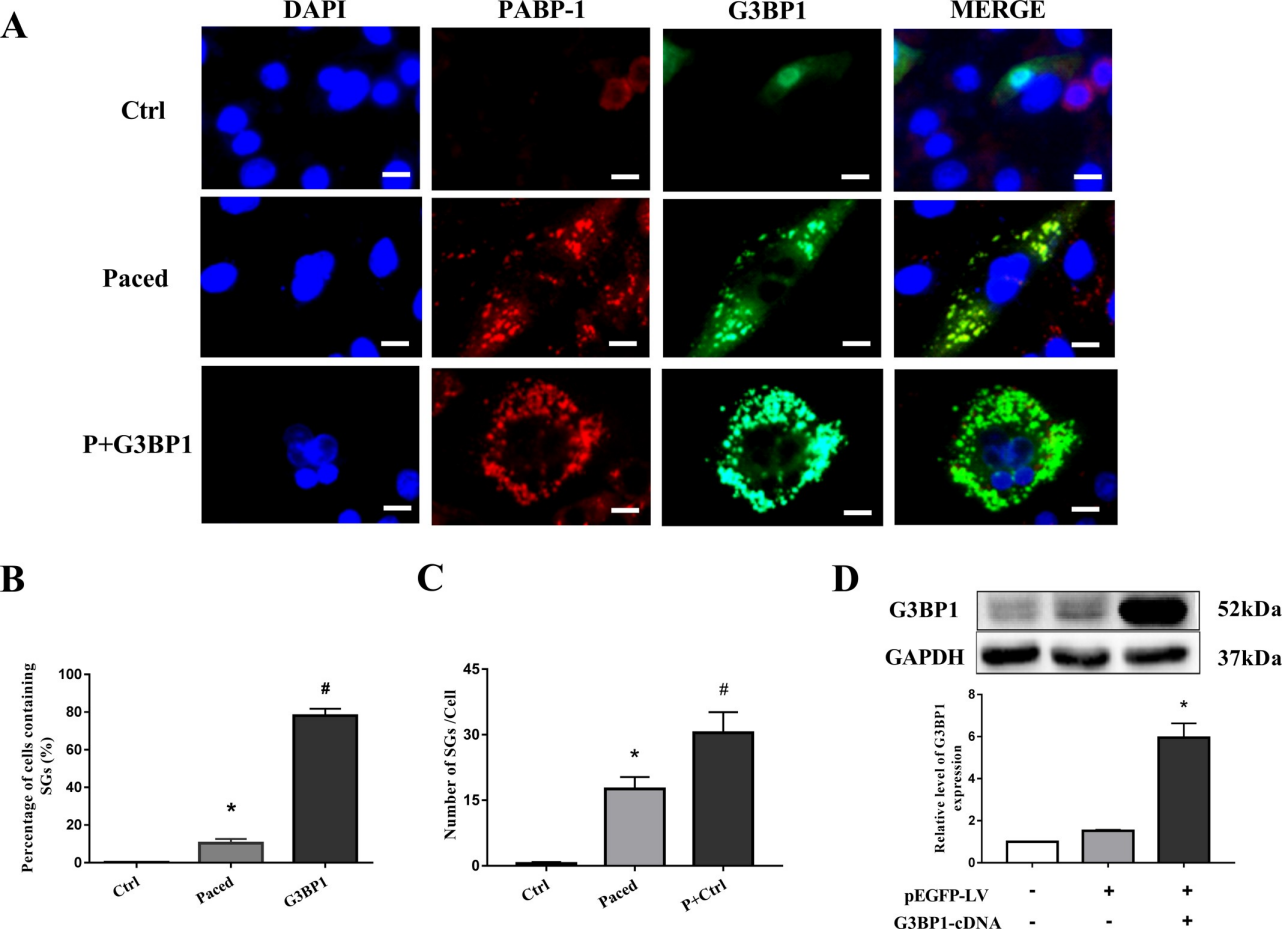
SGs were induced by pacing and G3BP1 overexpression in the stable HL-1 cell line. (A) Immunofluorescence was used to visualize SGs, which were indicated by G3BP1 protein (green) and endogenous PABP-1 protein (red), as well as their colocalization. Ctrl group: HL-1 cells; Paced group: cells stably expressing control plasmid that were paced for 6 h; and P+G3BP1 group: cells stably expressing G3BP1 that were paced for 6 h (scale bar is 100 μm). (B) Percentage of cells containing SGs (%), n = 5. *P < 0.05 compared with the ctrl group, ^#^P < 0.01 compared with the paced group. (C) Number of SGs in the 3 groups, n = 6. *P < 0.01 compared with the Ctrl group, ^#^P < 0.05 compared with the Paced group. (D) The expression of G3BP1 protein as detected by western blot was quantified; n = 4, *P < 0.01 compared with the pEGFP-LV group. All data are expressed as the mean ± SEM.

To investigate the function of SGs in AF, HL-1 cells were transfected with exogenous G3BP1 to increase SG formation. SGs were double-labeled with both G3BP1 and PABP-1, as indicated by green fluorescence and red fluorescence signals. Consistent with the above results, pacing significantly increased the formation of SGs (10.4±2.3% vs 0.2±0.2%, P<0.05), although the formation is partial and at a low level (Fig 2B). As expected, G3BP1 overexpression further increased the formation of SGs (Fig 2A–2C). Fig 2C shows the average number of SGs contained in each HL-1 cell and that the number of SGs is increased by G3BP1 overexpression. Furthermore, a cell line that stably overexpressed G3BP1 was successfully constructed. The expression of G3BP1 protein in the cell line was detected by western blot, and G3BP1 overexpression was confirmed (Fig 2D).

### Exogenous G3BP1 reduce oxidative stress and calcium overload in paced HL-1 cells

**Fig 3:**
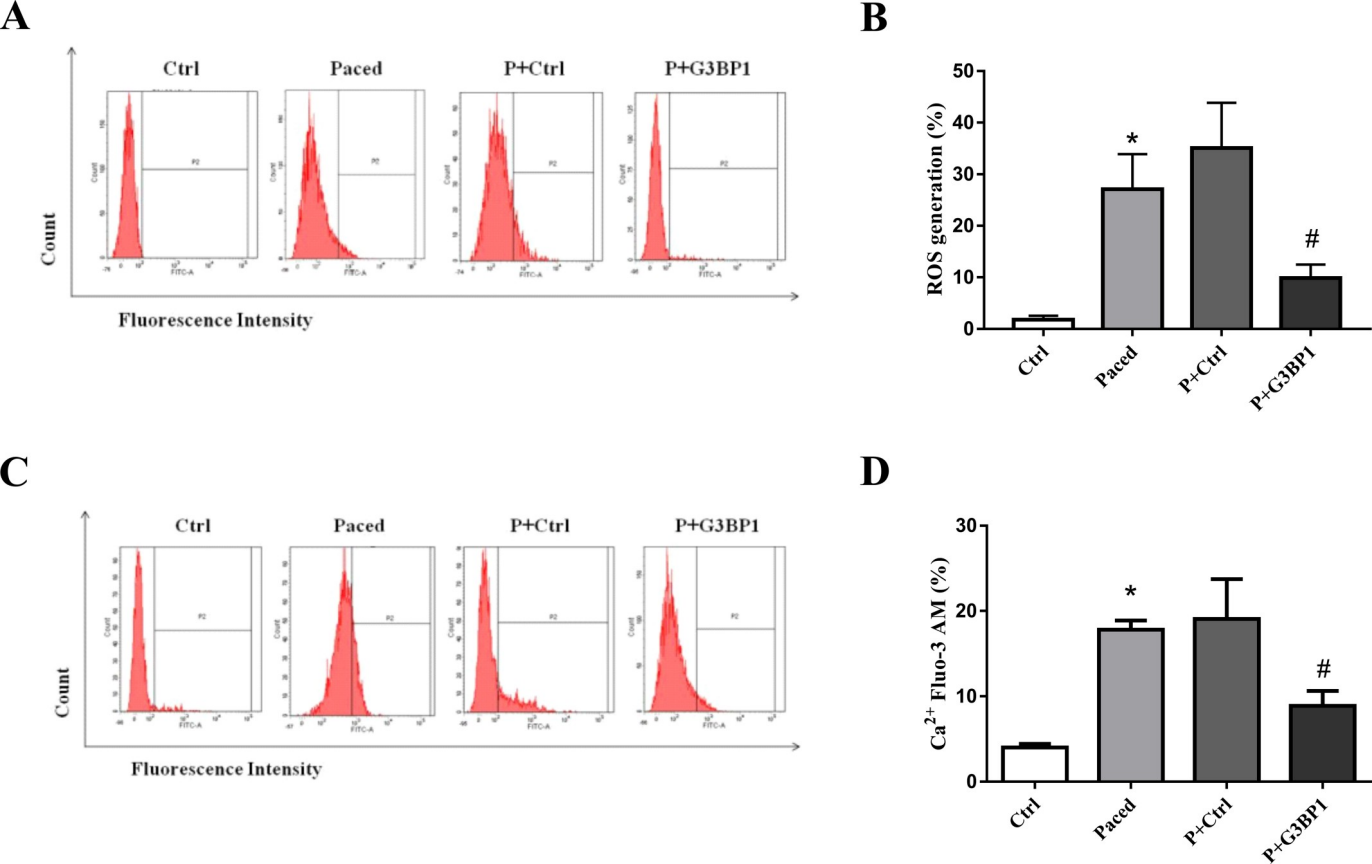
Effect of exogenous G3BP1 on ROS production and calcium overload in the paced stable HL-1 cell line. Flow cytometry was used to analyze the level of ROS (A) and the amount of Ca^2+^ (C) in HL-1 cells, n = 6. (B,D) Quantification of relative ROS and Ca^2+^ levels; n = 6. Data are expressed as the mean ± SEM. Ctrl: HL-1 cells; Paced: HL-1 cells paced for 6 h; P+Ctrl: cell lines stably expressing control plasmid that were paced for 6 h; and P+G3BP1: cell lines stably expressing G3BP1 that were paced for 6 h. *P < 0.01 compared with the Ctrl group; ^#^ P < 0.05 compared with the P+Ctrl group.

There was a significant increase in ROS level in rapidly paced atrial cells compared to that in the control group. The level of ROS was significantly decreased in paced HL-1 cardiomyocytes transfected with pEGFP-LV-G3BP1 compared to paced cells transfected with pEGFP-LV-con (Fig 3A and 3B), which suggests that G3BP1 overexpression can inhibit the oxidative stress caused by AF. To identify the effects of exogenous G3BP1 on calcium overload, cytosolic free Ca^2+^ was measured by flow cytometry. Flow cytometry showed that pacing significantly enhanced the Ca^2+^ concentration in HL-1 cells and that exogenous G3BP1 significantly decreased the level of Ca^2+^ in paced HL-1 cells (Fig 3C and 3D).

### G3BP1 overexpression prevent collagen expression in AF

**Fig 4:**
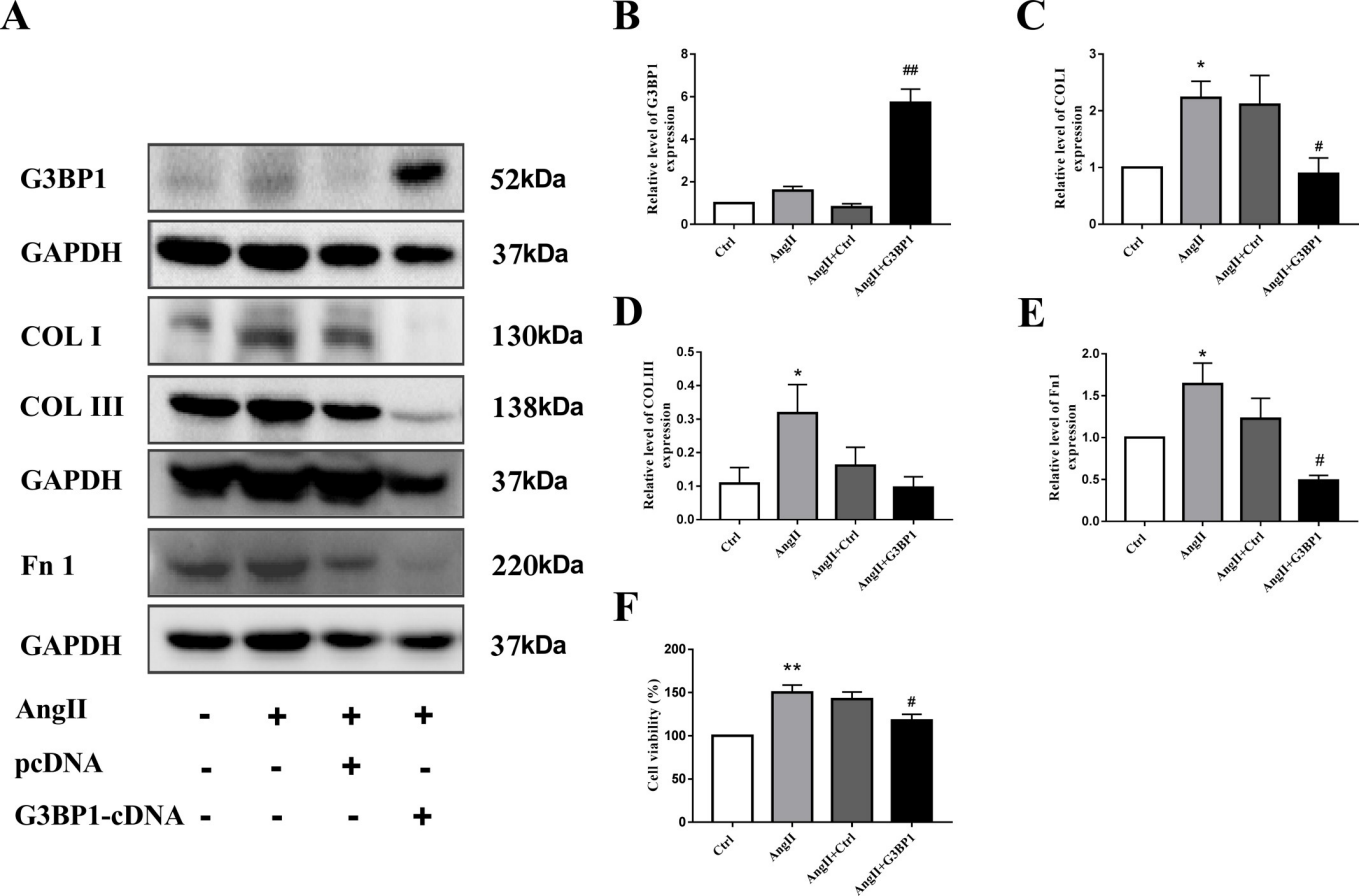
G3BP1 prevent AngII-induced cardiac fibroblast proliferation and collagen synthesis. A, Proteins were detected by western blot. B-E, Data are expressed as the mean ± SEM (n = 6). F. The CCK8 method was used to analyze the proliferation of cardiac fibroblasts induced by AngII and/or transient transfection of G3BP1 cDNA. *P < 0.05 compared with the control group; ^#^P < 0.05 compared with the AngII+Ctrl group. **P < 0.01 compared with the control group; ^##^P < 0.01 compared with the AngII+Ctrl group.

There is growing evidence that atrial fibrosis plays a vital role in the mechanism of atrial fibrillation. To determine whether G3BP1 overexpression impact fibrosis in AF, we overexpressed G3BP1 in cardiac fibroblasts. The overexpression of G3BP1 protein was evidenced by western blot (Fig 4A and 4B). AngII can induce the proliferation of fibroblasts, while G3BP1 overexpression significantly inhibit the proliferation of cardiac fibroblasts induced by AngII stimulation (Fig 4F). To further elucidate the effect of G3BP1 on atrial fibrosis in AF, we examined the effect of G3BP1 on collagen expression. The results showed that G3BP1 can inhibit the expression of COL I and FN1 (Fig 4C and 4E).

### Protein synthesis in AF

**Fig 5:**
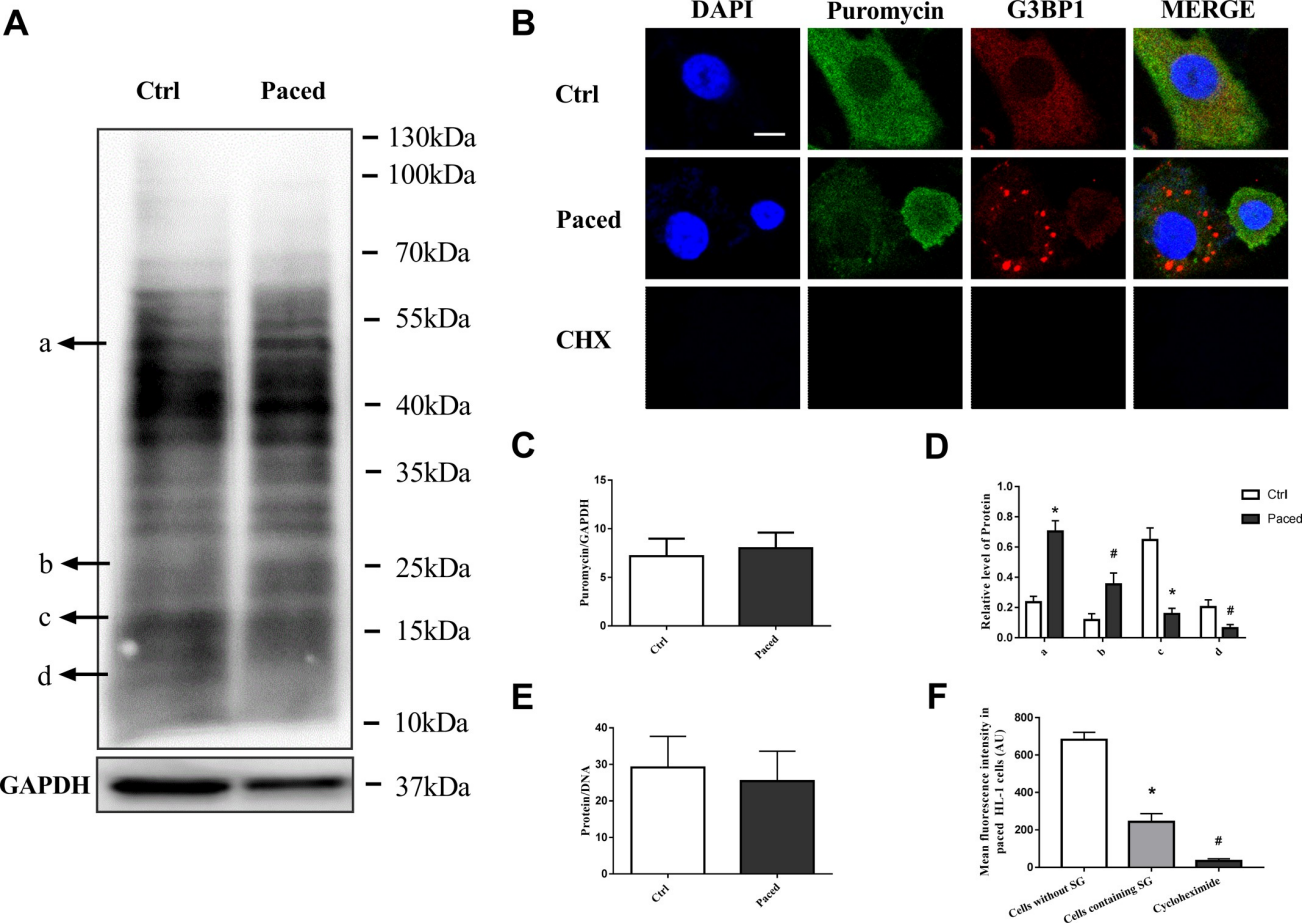
Protein synthesis in paced HL-1 cells. A, Puromycin was incorporated into HL-1 cells and proteins were detected by western blot. B, Puromycin was incorporated into HL-1 cells and protein synthesis in single cells was analyzed by immunofluorescence. Puromycin was indicated by green fluorescence and G3BP1 was indicated by red fluorescence signals. C,D The amount of protein is expressed as the mean ± SEM (n = 6). *P < 0.01 compared with the control group; ^#^P < 0.05 compared with the control group. E, The ratio of protein to DNA (n = 6). F, Mean fluorescence intensity in HL-1 cells was shown as mean ± SEM (n = 6). *P < 0.01 compared with the cells without SG, ^#^P < 0.01 compared with the cells containing SG.

SGs typically form when global protein synthesis is suppressed. To determine whether there is a general inhibition of protein synthesis in AF, puromycin incorporation was used in paced HL-1 cells. The results showed that pacing did not cause a general inhibition of protein synthesis (shown in Fig 5A and 5C). In pacing HL-1 cells, the synthesis of some proteins increased (protein a, b in Fig 5A and 5D), and the synthesis of other proteins decreased (protein c, d in Fig 5A and 5D). The ratio of protein to DNA was calculated and the results also indicated that there was no universal protein inhibition in AF (Fig 5E). These results seemed to be inconsistent with most circumstances, under which SGs are tightly linked to a suppression of translation. Therefore, we further assessed the protein synthesis rate in single cells by immunofluorescence analysis. We found that puromycin incorporation in SG-containing cells significantly reduced compared with that in cells without SGs (P<0.01) (Fig 5B and 5F).

## Discussion

In the present study, we demonstrated for the first time that there were SGs in cardiomyocytes following AF. We transfected the exogenous G3BP1 overexpression plasmid into HL-1 cells and observed the formation of SGs and a series of morphological changes in SGs under different pacing times. Then, we demonstrated that G3BP1 overexpression not only promoted the formation of SG, reduced ROS accumulation and calcium overload but also inhibited fibrogenesis induced by AngII stimulation. G3BP1 overexpression can also reduce cell proliferation and inhibit the expression of COLI and FN1. This finding may play an important role in further studies of the pathophysiological mechanisms and treatments of AF.

### SGs induced by ROS in AF

AF is the most common arrhythmia in the clinic, but limited treatments are available for AF. Emerging evidence suggests a significant role for oxidative stress, which has been demonstrated to be an inducer of SG formation [11], in the pathogenesis of AF [14–16]. On the one hand, rapid cardiac pacing was equivalent to a strong oxidative stimulus, increasing myocardial oxidative stress [17]. Studies in humans and animals have demonstrated that increased ROS production is present in cardiac specimens during AF [15,18]. Additionally, there was a significant increase in ROS production in rapidly pacing atrial cells in the present study. On the other hand, oxidative stress and ROS overproduction in AF may in turn induce SG formation. Studies have suggested that oxidative stress reagents, such as sodium arsenite [19] and hydrogen peroxide [20], strongly induce SG formation in mammals. In yeast cells, ROS production can also trigger SG formation [21]. Thus, rapid atrial frequency during AF induces oxidative stress and excess ROS, which may further induce the formation of SGs in the heart. However, unlike most circumstances, only a small proportion of cells formed SGs in paced cardiomyocytes, which can be explained by only low-grade inflammation was present in AF [22], and thus the formation of SGs in AF was partial and at low-level.

#### G3BP1 overexpression decrease ROS in AF

Sustained high levels of ROS production cause enormous damage to cells and tissues, such as inflammation, massive cell death, and aging [23]. To defend the organism against such injuries, cells rapidly activate a series of antioxidant mechanisms. We found that G3BP1 overexpression exhibits antioxidant activity during the AF-induced stress response and can inhibit the structural remodeling of cells. The beneficial effects of G3BP1 overexpression may be explained by two possibilities. One of them is that the induction of SG formation by G3BP1 overexpression in HL-1 cells could significantly reduce pacing-induced ROS accumulation and counteract the oxidative stress induced by AF, which is consistent with previous studies [8,9]. The other is that the antioxidant effects observed by G3BP1 overexpression could be a direct consequence of elevated G3BP1 levels. A study has demonstrated that nuclear G3BP1 inhibited the maturation of primary miR-15b~16–2 and miR-23a~27a~24–2 to [24], and thus contributed to inflammation inhibition [25].

#### G3BP1 decrease Ca^2+^ overload in AF

Ca^2+^ overload plays a crucial role in the pathophysiological process of AF. The onset of AF is thought to be the result of calcium overload because conditions that increase atrial calcium load increase the frequency of spontaneous mechanical activity and thus trigger atrial tachyarrhythmias [26]. Studies have suggested that calcium overload can damage atrial myocytes and initiate electrophysiological and structural remodeling in AF [26–28]. Therefore, reducing calcium overload may prevent AF development, which is one of the important measures for the treatment of AF. In this study, flow cytometry was used to confirm that G3BP1 overexpression can effectively reduce calcium overload in rapidly paced atrial cells, showing that G3BP1 overexpression may reverse the corresponding pathological process of calcium overload in AF. This result is consistent with research findings that show calcium channel blockade leads to a more sustained effect in preventing atrial electrical remodeling [27]. The exact mechanism remains unclear and may be related to the ability of G3BP1 to inhibit ROS production, further attenuate Ca^2+^ overload and prevent triggering arrhythmic activity [28].

#### G3BP1 decrease collagen expression in AF

ROS are known to play a key role in fibrosis and the induction of AF. Clinical and animal studies have demonstrated that ROS alter multiple cardiac ionic currents and impair gap junction function, resulting in reduced myocyte coupling and facilitation of reentry. Excessive production of ROS is likely involved in the structural and electrical remodeling of AF, contributing to fibrosis, during which NADPH oxidase (NOX) has emerged as a potential enzymatic source for ROS production in AF [29]. We also demonstrated previously that ROS enhance the proliferation of fibroblasts and increase the protein expression of collagen and fibronectin-1 in AngII-stimulated cardiac fibroblasts [18]. Thus, suppression of oxidative stress and ROS by G3BP1 overexpression may have a potential beneficial role in the prevention and treatment of AF.

#### Limitations

First, although we observed that SGs are present in paced HL-1 cells and in cardiomyocytes, this presence was partial and at a low level, and insufficient to perform a beneficial effect. We overexpressed G3BP1 to increase SGs formation, but the G3BP1 protein itself was also increased, so we cannot conclude the effects observed by G3BP1 overexpression were a consequence of elevated numbers of SGs or a direct consequence of G3BP1 overexpression. Second, this study was mainly performed on HL-1 cells. The electrophysiological, functional, and metabolic properties of HL-1 cells are modified during culture, although their cardiomyocyte identity has been verified. Third, we did not detect the components of SGs and which proteins were recruited into SGs under the pathological conditions of AF. Fourth, we did not dynamically observe the effects of SG aggregation and depolymerization on ROS, calcium overload, and fibrosis in AF. ROS scavengers should be employed to better evaluate the impact of ROS on SGs formation.

## Conclusion

In summary, we were the first to discover that SGs exist in AF myocytes although the presence was partial and at a low level. At the same time, we also found that G3BP1 overexpression had an inhibitory effect on ROS aggregation and calcium overload, as well as collagen expression under stress conditions in AF. The dual effects of G3BP1 on electrical and structural changes induced by AF in myocardial cells will provide a new perspective for the treatment of AF.
